# Mechanical and Dielectric Properties of Two Types of Si_3_N_4_ Fibers Annealed at Elevated Temperatures

**DOI:** 10.3390/ma11091498

**Published:** 2018-08-22

**Authors:** Jie Zhou, Fang Ye, Xuefeng Cui, Laifei Cheng, Jianping Li, Yongsheng Liu, Litong Zhang

**Affiliations:** Science and Technology on Thermostructural Composite Materials Laboratory, Northwestern Polytechnical University, Xi’an 710072, China; 13628013651@163.com (J.Z.); yefang511@nwpu.edu.cn (F.Y.); cuixuefeng@nwpu.edu.cn (X.C.); ijianping@163.com (J.L.); yongshengliu@nwpu.edu.cn (Y.L.); zhanglt@nwpu.edu.cn (L.Z.)

**Keywords:** Si_3_N_4_ fiber, wave-transparent, thermal stability, mechanical properties, dielectric properties

## Abstract

The mechanical and dielectric properties of two types of amorphous silicon nitride (Si_3_N_4_) fibers prior to and following annealing at 800 °C were studied. The tensile strengths of the Si_3_N_4_ fiber bundles were measured using unidirectional tensile experimentation at room temperature, whereas the permittivity values were measured at 8.2–12.4 GHz using the waveguide method. The results demonstrated that the tensile strength and dielectric properties of Si_3_N_4_ fibers were correlated to the corresponding composition, microstructure, and intrinsic performance of electrical resistance. The Si_3_N_4_ fibers with a lower content of amorphous SiN_x_O_y_ presented an improved thermal stability, a higher tensile strength, a higher conductivity, and a significantly stable wave-transparent property. These were mainly attributed to the highly pure composition and decomposition of less amorphous SiN_x_O_y_.

## 1. Introduction

Radome [1,2], as an indispensable part of high-performance missile weapons, plays a vital role in the normal operation of these weapons. In order that the missile weapons function in harsh environments, the performance required for wave-transparent materials under high temperature is increasingly demanding [3,4,5,6]. Silicon nitride (Si_3_N_4_), which demonstrates high strength, good thermal shock resistance, wear resistance, outstanding oxidation resistance, and chemical stability characteristics, is increasingly studied and applied as a high-temperature structural ceramic [7,8,9,10,11]. In addition, Si_3_N_4_ ceramics possess a low dielectric constant and a high electrical resistivity, which are the preferred properties for the transmission and insulation of electromagnetic waves in high-temperature environments [12,13,14,15,16]. Furthermore, Si_3_N_4_ nanomaterials, another hotspot of research, have both the advantages of Si_3_N_4_ and nanomaterials, which could be used to prepare nanofluids possessing different properties than their solids [17,18]. The continuous Si_3_N_4_ ceramic fiber is a new type of high-performance ceramic fiber, developed on the basis of precursor-converted continuous SiC ceramic fibers [19,20,21]. As a type of Si_3_N_4_ material, it has a series of excellent properties, proving to be a popular reinforced material candidate for high-temperature wave-transparent ceramic matrix composites (CMCs) used in radomes [21,22].

Considering the Si_3_N_4_ fiber as a core reinforcement material for radomes, there are strict requirements to follow regarding the mechanical and dielectric properties. Ideally, low and stable dielectric constant (ε) and dielectric loss (tan δ) are required in addition to the necessary mechanical properties, generally with values not exceeding 4.0 and 0.01 [23,24], respectively. During preparation, the fiber composition can differ due to different conditions, which affect it directly, consequently affecting the corresponding microscopic structure and performance [25,26]. In this study, two types of Si_3_N_4_ fibers were heat treated at 800 °C, while the composition, microstructure, tensile strength, complex permittivity (ε_r_ = ε′ − ε″j), and dielectric loss (tan δ = ε″/ε′) of both Si_3_N_4_ fibers, in the as-received and heat-treated states, were investigated in detail.

## 2. Experimentation

### 2.1. Materials and Heat Treatments

The materials selected for experimentation were two different types of Si_3_N_4_ fiber bundles, supplied by Xiamen University of China, each consisting of 500 filaments, and woven into two-dimensional cloths by the Shaanxi Institute of Textile Science. The as-received Si_3_N_4_ fibers were produced by the pyrolysis of polycarbosilane (PCS). Because of the different pyrolysis conditions during preparation, the test results showed that the most significant difference between the two types of fibers was the oxygen content. For the convenience of subsequent description, the Si_3_N_4_ fiber with the relatively low oxygen content of 3.56 wt % (determined by a CONS elemental analyzer) was termed as Si_3_N_4_ fiber-L; the Si_3_N_4_ fiber with the relatively high oxygen content of 13.56 wt %, corresponded to Si_3_N_4_ fiber-H. In order to analyze the preparation temperature of the composites toughened by the Si_3_N_4_ fibers to see whether it had an effect on the characteristic performance of the fibers, the Si_3_N_4_ fibers and fiber cloths were placed in a furnace for chemical vapor decomposition (CVD) without gas at the temperature of 800 °C for up to 2 h of heat treatment, to simulate the preparation environment [27].

### 2.2. Microstructure Characterization

The surface and cross-section morphology of the Si_3_N_4_ fibers were analyzed using scanning electron microscopy (SEM) (S4700, Hitachi, Tokyo, Japan). The phase compositions of the Si_3_N_4_ fibers were analyzed using X-ray diffraction (XRD) (X’ Pert Pro, Philips, Amsterdam, The Netherlands) with Cu Kα (λ = 1.54 Å) radiation. The surface composition and chemical bonding states of the Si_3_N_4_ fibers were measured using X-ray photoelectron spectroscopy (XPS) (Axis Ultra, Oxford, UK). The fiber microstructure and crystalline state were accurately described using transmission electron microscopy (TEM) (G-20, FEI-Tecnai, Hillsboro, OR, USA). The heat treatment behavior of the as-received fibers was determined using thermogravimetric (TG) and differential scanning calorimeter (DSC) analysis (STA 449C, Selb, Germany) under Ar atmosphere at the heating rate of 10 °C/min to a maximum of 800 °C.

### 2.3. Property Characterization

**Figure 1:**
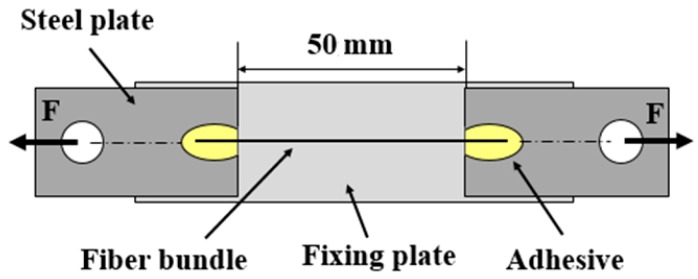
Schematic illustration of tensile specimen for single fiber bundles.

The unidirectional tensile strengths at room temperature of the fiber bundles were tested. The two ends of the bundles were fixed on a steel plate with an adhesive of 50 mm in gauge length and of 0.2 mm/min in cross-head draw speed. This process was sufficiently slow, to simulate a quasi-static loading. Figure 1 presents the schematic illustration of a tensile specimen for single fiber bundles.

The electrical resistance of fiber bundles was tested with DC sources (6220, Keithley, Cleveland, OH, USA). Silver paste was added as coating on both ends of the fiber bundles to ensure a good electrical contact. The voltage values were determined by the corresponding current values, whereas the average value of resistance (*R*) was obtained using Ohm’s law. The average resistivity of each bundle of fibers (μ) was calculated through the following equation:

(1) $$\mu \;=\;\frac{RS}{l},$$

where *S* and *l* are the cross-sectional area and length of the fiber bundles, respectively. The relative complex permittivity testing of Si_3_N_4_ fibers was conducted using a vector network analyzer (VNA) (MS4644A, Anritsu, Atsugi, Japan), with the waveguide method in the frequency band of 8.2–12.4 GHz. To ensure that the Si_3_N_4_ fibers could be fixed to facilitate the measurement of dielectric property, the Si_3_N_4_ fabrics, produced from Si_3_N_4_ fiber-L and Si_3_N_4_ fiber-H, were respectively composited with epoxy resin (68 vol %) to obtain fiber/resin composites (named as samples 1 and 2, respectively). To prevent the corresponding effects on the conductivity and dielectric properties, the surface sizing agents on both types of original Si_3_N_4_ fibers were first removed by water soaking at 80 °C for a period longer than 0.5 h.

## 3. Results and Discussion

### 3.1. Microstructure and Composition of As-Received Fibers

Table 1 presents the fundamental characteristics of the Si_3_N_4_ fibers. The O and C contents of the Si_3_N_4_ fiber-H were higher, whereas the corresponding mechanical properties were closer to the precursor state [28].

**Figure 2:**
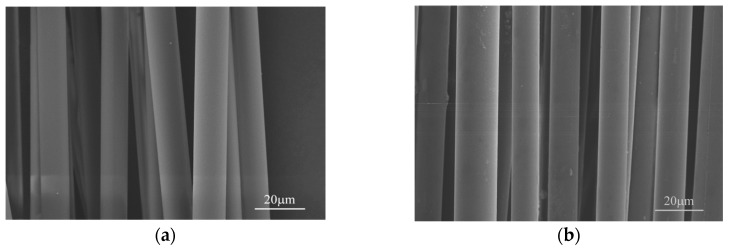

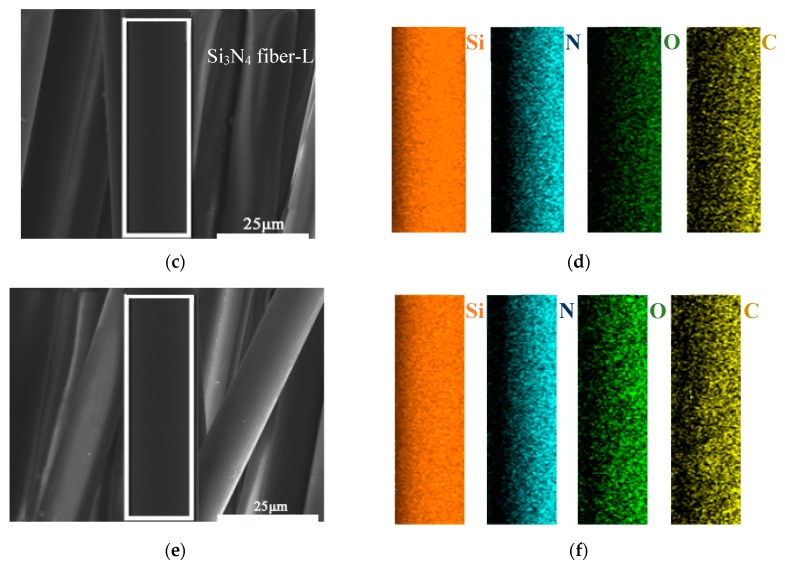
(**a**) Surface SEM images of Si_3_N_4_ fiber-L and (**b**) Si_3_N_4_ fiber-H, (**c**,**d**) scanning electron microscopy and energy dispersive pectroscopy (SEM-EDS) element mapping of Si_3_N_4_ fiber-L and (**e**,**f**) Si_3_N_4_ fiber-H.

Figure 2 presents the surface morphology and element mapping of both desized Si_3_N_4_ fibers. The surfaces of both fibers (see Figure 2a,b) were smooth and flat without apparent defects, with no distinct difference between them. It was observed that both fibers displayed relatively uniform diameters of approximately 12.2 μm and 13 μm, respectively. The element mapping (see Figure 2c–f) presented the uniform distributions of Si, N, O, and C.

**Figure 3:**
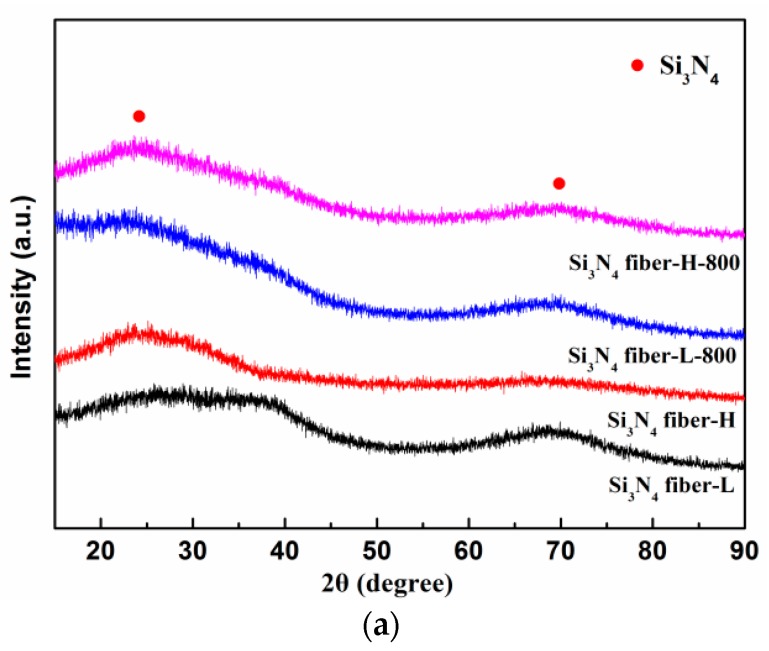

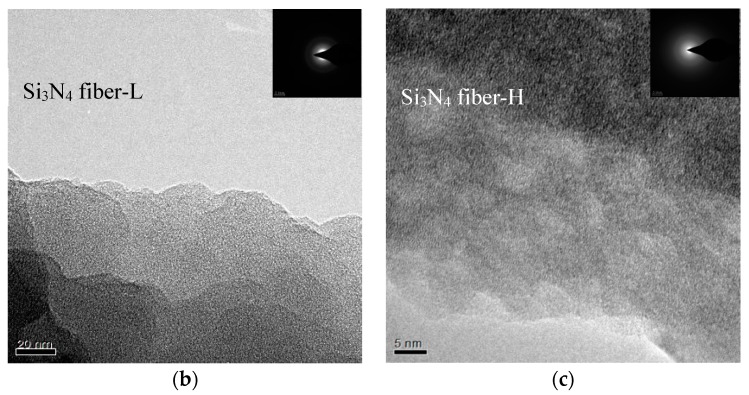
(**a**) X-ray diffraction (XRD) patterns of Si_3_N_4_ fibers prior to and following heat treatment and (**b**,**c**) transmission electron microscopy (TEM) patterns of untreated fibers.

The crystalline states of the untreated fibers and the heat-treated fibers at 800 °C are presented in Figure 3. No apparent crystal absorption peak were noted and only two broad diffraction peaks at approximately 23° and 69° were found in the typical XRD patterns of the two Si_3_N_4_ fibers annealed at 800 °C under vacuum. This revealed that the heat-treated fibers at 800 °C did not reach the crystallization temperature, remaining in the original amorphous state. High-resolution transmission electron microscopy (HR-TEM) combined with selected-area electron diffraction (SAED) were used to observe the detailed microstructures of both untreated Si_3_N_4_ fibers.

**Figure 4:**
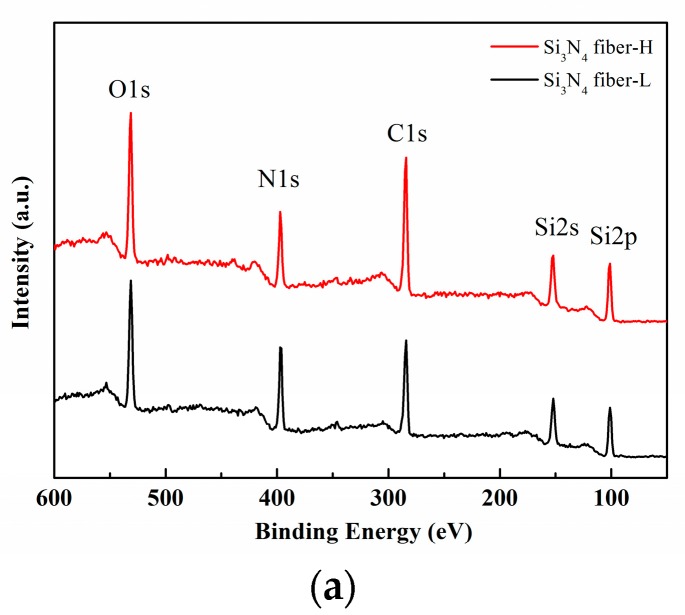

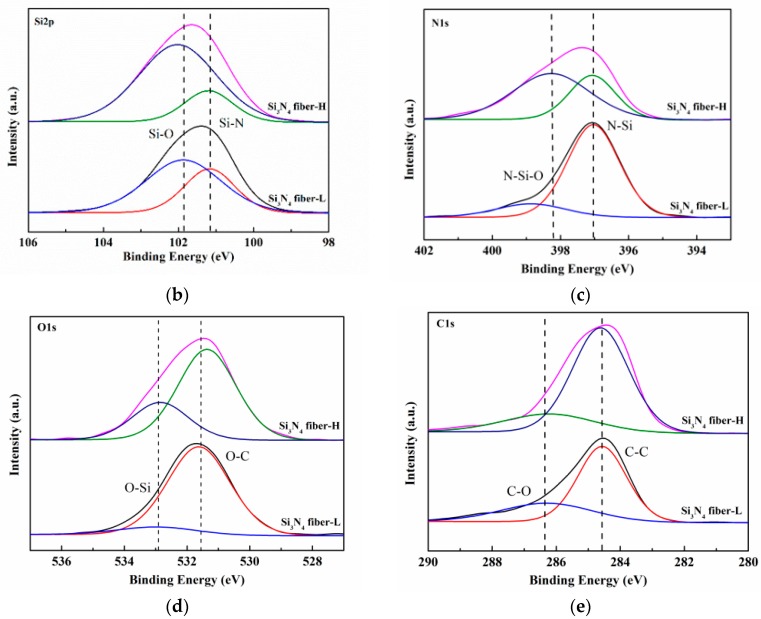
(**a**) XPS survey spectra, (**b**) Si 2p, (**c**) N 1s, (**d**) O 1s, and (**e**) C 1s core level spectra recorded from two desized fibers.

The XPS analyses of both desized fibers were performed to determine the corresponding surface composition and chemical bonding state. In the survey XPS spectra, presented in Figure 4a, the Si 2s, Si 2p, N 1s, C 1s and O 1s peaks were detected. As presented in Figure 4b, two peaks existed in the Si 2p spectrum: one peak was located at 101.8 eV and could be attributed to the Si–O bonds in the SiOx and SiN_x_O_y_ phases, whereas the other peak was located at 101.2 eV, due to Si_3_N_4_, where the Si–O bond occupied a higher proportion on the Si_3_N_4_ fiber-H surface compared to the Si_3_N_4_ fiber-L. Similarly, two peaks existed in the N 1s spectrum, as presented in Figure 4c: one peak was located at 397.0 eV and could be attributed to the N–Si bond, whereas the other peak was located at 398.2 eV due to the N–Si–O bond, which was offered by the SiN_x_O_y_ phase [29]. Furthermore, the O 1s spectrum presented in Figure 4d displayed two peaks. The lower energy peak was located at 532.9 eV, which corresponded well with the O–Si bond value. In addition, the higher energy peak located at 531.6 eV indicated the existence of COx at the fiber surface. At the Si_3_N_4_ fiber-H surface, a higher proportion of O–Si bond existed from the SiN_x_O_y_ phase. In Figure 4e, C–C bonds, corresponding to the peak located at 284.6 eV, and C–O bonds existed for both fibers. The peak was located at 286.2 eV, where the two bonds originated from the surface sizing agent on the fibers. Considering the existence of NH_3_ in the fiber preparation environment, the possibility of a SiN_x_O_y_ phase is much higher than that of a SiO_x_ one, following Reactions (2) and (3) [30]. Based on the latter analysis, it could be concluded that the two fibers were mainly composed of amorphous Si_3_N_4_, with low amounts of SiO_x_ and SiN_x_O_y_ phases.

(2) $${\mathrm{SiO}}_{2}{(s)\;+\;H}_{2}{(g)\;=\;\mathrm{SiO}(g)\;+\;H}_{2}O(g),$$

(3) $${\mathrm{SiO}(g)+\mathrm{NH}}_{3}{(g)\;=\;\mathrm{SiN}}_{x}O_{y}{(g)\;+\;H}_{2}O\left(g\right)+H_{2}(g),$$

### 3.2. Effects of Heat Treatment on Microstructure and Composition

The element content of both Si_3_N_4_ fibers following annealing are presented in Table 2. Compared with Table 1, the mass percentage of N elements in both fibers decreased significantly after heat treatment, which may be due to the SiN_x_O_y_ phase decomposition, following Reaction (4) [31].

(4) $${\mathrm{SiN}}_{x}O_{y}{(s)\;=\;\mathrm{SiO}(g)\;+\;N}_{2}(g),$$

**Figure 5:**
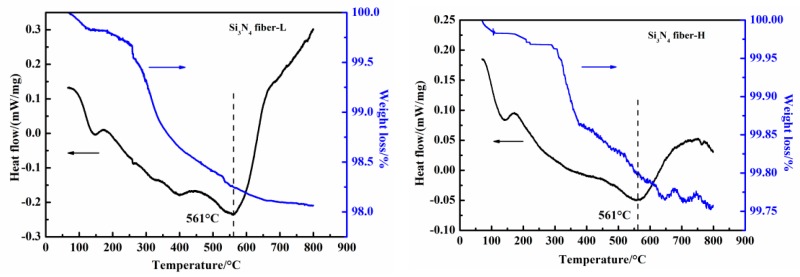
Thermogravimetric and differential scanning calorimeter (TG-DSC) plots of two as-received fibers under Ar atmosphere.

The TG-DSC plots of the two as-received fibers are presented in Figure 5. For both fibers, endothermic peaks appeared at approximately 561 °C along with a weight decrease prior to reaching that temperature, which corresponded to the sizing agent decompositions on the fiber surfaces. Adversely, the heat flow curve significantly dropped at approximately 750 °C and its quality had a downward trend for the Si_3_N_4_ fiber-H. This meant that another endothermic reaction would start to occur, which could be associated with the SiN_x_O_y_ phase decomposition. Because of the negligible content during the SiN_x_O_y_ phase, the SiN_x_O_y_ decomposition caused almost no impact on the Si_3_N_4_ fiber-L.

**Figure 6:**
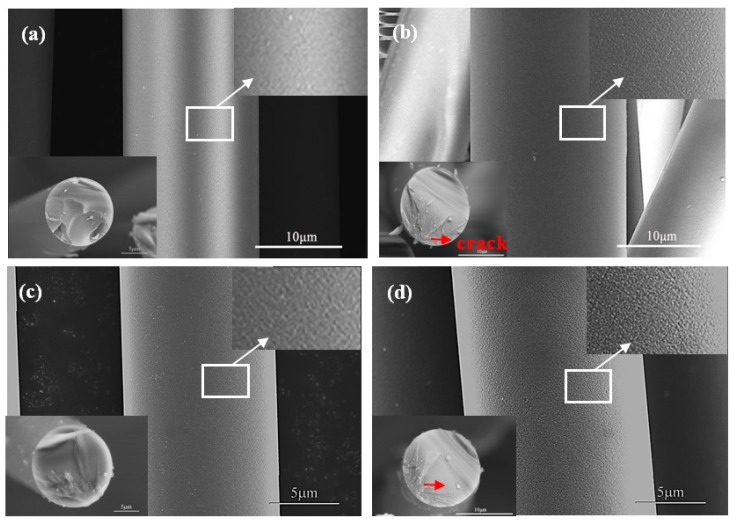
Surface and cross-section (insets) morphologies of (**a**,**b**) Si_3_N_4_ fiber-L and (**c**,**d**) Si_3_N_4_ fiber-H prior to and following heat treatment.

The surface and cross-section morphology (insets) of the two types of desized fibers prior to and following heat treatment are presented in Figure 6, respectively. It was clear that the effect of heat treatment at 800 °C on the microstructure could be observed. Following heat treatment, the surfaces of both fibers changed from smooth and flat to rough. This occurred mainly due to the SiN_x_O_y_ decomposition, affecting the fibers’ performances. Moreover, a typical brittle fracture model could be observed from the cross-section morphology. The characteristic mirror, mist, and hackle features were clearly evident in these micrographs [32]. From the crack propagation path, it was observed that the crack source was mainly from the surface flaws for the heat-treated fibers.

**Figure 7:**
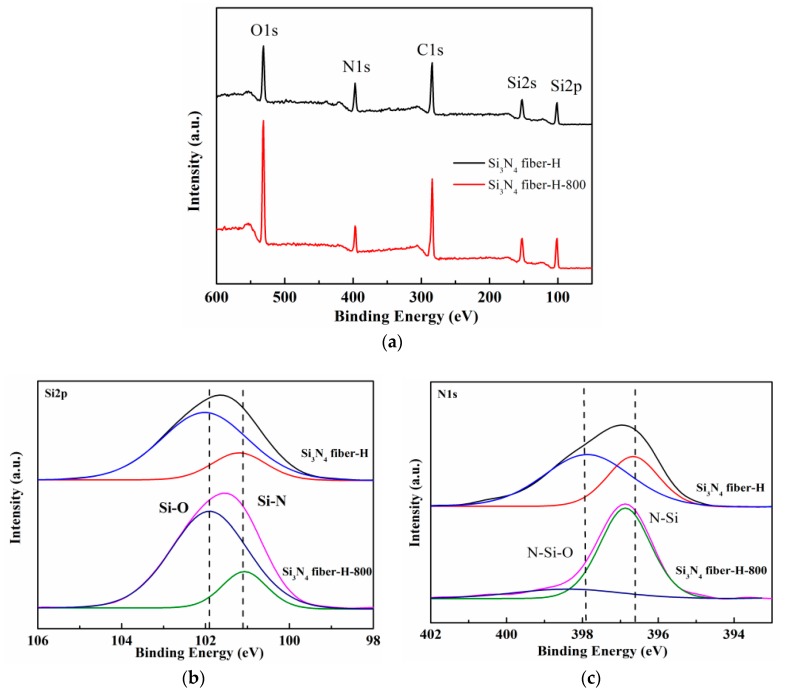
(**a**) XPS survey spectra, (**b**) Si 2p, and (**c**) N 1s core level spectra recorded from Si_3_N_4_ fiber-H prior to and following annealing.

In order to verify the SiN_x_O_y_ phase decomposition at the Si_3_N_4_ fiber-H surface, the corresponding surface chemical bonding states prior to and following annealing were analyzed using XPS. The (a) survey XPS spectra, (b) Si 2p, and (c) N 1s core level spectra are presented in Figure 7. Compared to the initial Si_3_N_4_ fiber-H, it was clear that the ratio of Si–O bond to Si–N bond had almost no change following annealing. This occurred because the heat treatment at 800 °C had little effect on the SiO_x_ phase. In contrast, the N–Si–O bond had a significant reduction following annealing, as the main reason for the amount increase in surface defects, which proved the SiN_x_O_y_ phase decomposition.

### 3.3. Effect of Heat Treatment on Room-Temperature Tensile Strength

**Figure 8:**
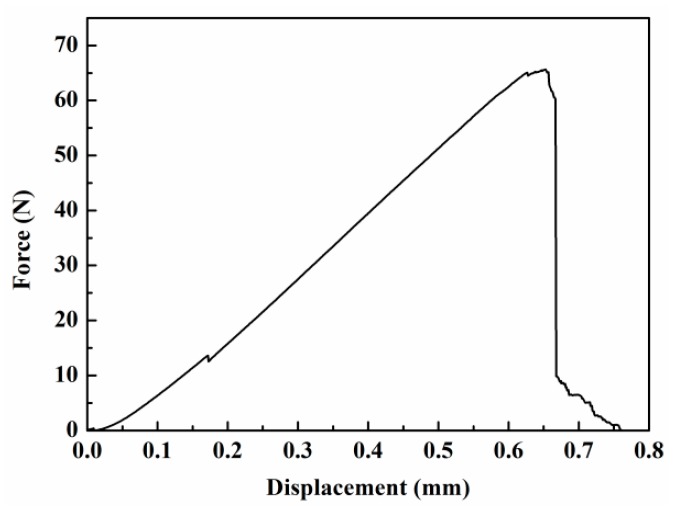
Typical experimental load-displacement curve of the fiber bundles, presenting simultaneous fracture behaviors.

The tensile strengths of fibers were measured using the fiber bundle tensile strength measurements. The corresponding dispersions could be well described using the Weibull distribution with two parameters [29]. Figure 8 presents the typical experimental load-displacement curve of the fiber bundles. Figure 8 shows that the fiber bundles were elastically deformed before breaking and that the elastic modulus was constant. Almost all filaments followed a simultaneous fracture behavior when the load reached its maximum, proving that the fiber bundle testing was reasonable. Moreover, a drop point and a post-failure “tail” region were noted. The former was the shake result of the testing equipment, whereas the latter occurred due to friction among neighboring filaments failing at different relative positions. These were not considered for the Weibull parameter calculation in this paper [33].

The two-parameter Weibull function is given as follows [34]:

(5) $$F\;=\;1\;-\;\exp[-(\frac{\sigma_{i}}{\sigma_{0}}{)}^{m}],$$

where *F* is the fracture probability of the fiber under a uniaxial tensile stress σ*_i_*, which is defined as fracture strength. σ_0_ and *m* are the Weibull scale parameter and the Weibull modulus, respectively. As the value of *m* becomes higher, the strength distribution range becomes narrower and the function curve becomes steeper. Both depend only on the value of *m*.

The following formula can be obtained, after taking two times the logarithm of Equation (5):

(6) $$\mathrm{lnln}\left(\frac{1}{1\;-\;S}\right){\;=\;m\ln\sigma }_{i}\;-{\;m\ln\sigma }_{0},$$

where *S* is the fracture probability from experiments and can be defined according to Equation (7):

(7) $$S_{i}\;=\;\frac{i\;-\;0.5}{n},$$

where *n* is the sample number and *i* is the rank of σ*_i_*. When the sample size was approximately 10, the *S_i_* was in a weighted analysis [35]. It was apparent that the lnln(1/(1 − *S*)) and lnσ*_i_* had a linear correlation, in which the slope was equal to *m* and the intercept of lnln(1/(1 − *S*)) axis was equal to −*m*lnσ_0_. Therefore, the values of *m* and σ_0_ could be derived from the experimental data and graphical relationships between lnln(1/(1 − *S*)) and lnσ*_i_*.

Table 3 lists the Weibull statistics and tensile strength of the two types of Si_3_N_4_ fiber bundles annealed under vacuum.

**Figure 9:**
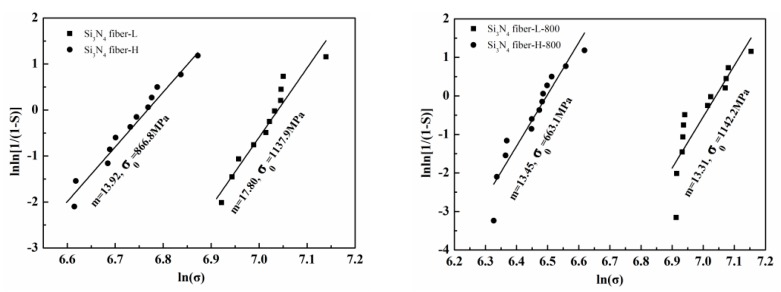
Weibull plots for fiber bundle tests with and without heat treatment.

Figure 9 presents the linear fitting of the relationship between lnln(1/(1 − *F*)) and lnσ*_i_* of the two Si_3_N_4_ fiber bundles. It was clear that the Weibull plots were evenly distributed on both sides of the fitted line, which indicated that the tensile strengths of the fiber bundles followed the two-parameter Weibull distribution.

**Figure 10:**
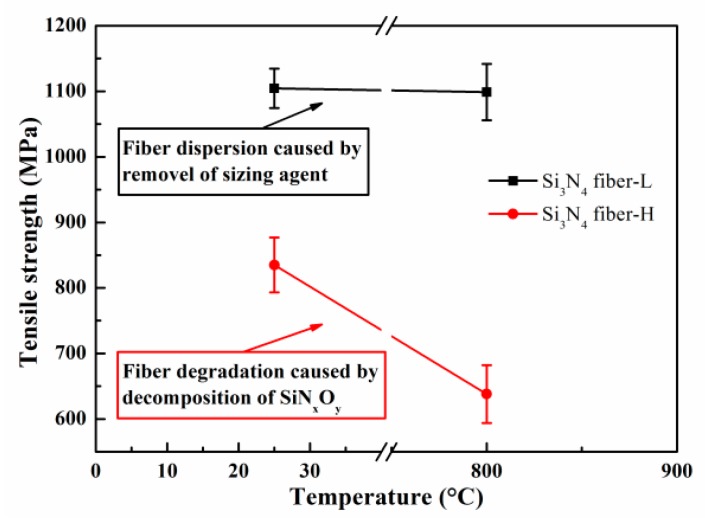
Single tow tensile test results.

Figure 10 presents the tensile test results combined with Table 3 and Figure 9. For the as-received fibers, the Si_3_N_4_ fiber-L strength was 1104.4 MPa, exceeding the 825 MPa reached by the Si_3_N_4_ fiber-H. In addition, compared to the Si_3_N_4_ fiber-H, the Si_3_N_4_ fiber-L had a higher Weibull modulus, which might be attributed to the neater arrangement and fewer internal flaws of filaments, where more filaments could evenly bear load and fracture at the same time, leading to higher strength and more centralized data. Following annealing at 800 °C under vacuum, the strength retention rate of the Si_3_N_4_ fiber-L was almost 100%, proving that the heat treatment at 800 °C had no effect on its structure, while the value of *m* decreased from 17.8 to 13.31. This occurred because the fiber bundles scattered and the filaments could be uniformly loaded, mainly due to the sizing agent removal on the fiber surface. The tensile experimentation results demonstrated that the Si_3_N_4_ fiber-L following heat treatment at 800 °C could still maintain good tensile properties. Adversely, the Si_3_N_4_ fiber-H tensile strength decreased from 835 MPa to 638 MPa, while the value of *m* was almost constant following heat treatment, demonstrating that the filaments of the as-received Si_3_N_4_ fiber-H had a high dispersion by nature. The strength retention result of only 76% indicated that the heat treatment at 800 °C caused damage due to the SiN_x_O_y_ phase decomposition, resulting in a roughened and more defective surface of the Si_3_N_4_ fiber-H microstructure. This was consistent with the information presented in Figure 6.

According to the research by Taylor [32], a relationship exists among the different diameters of fibers and their strength. As mentioned above, the Si_3_N_4_ fiber-H diameter was higher. Because of the size effect, the probability of existing defects in the Si_3_N_4_ fiber-H was higher, which led to a lower and more dispersive strength. Furthermore, a higher content of SiN_x_O_y_ phase was noted, resulting from the Si_3_N_4_ fiber-H impurities, which were unstable and would be destroyed, while bringing flaws and reducing performance at a high temperature. These could be indirectly proved from Figure 6, demonstrating that the Si_3_N_4_ fiber-L had improved mechanical properties and high thermal stability.

### 3.4. Effect of Heat Treatment on Room-Temperature Dielectric Properties

**Figure 11:**
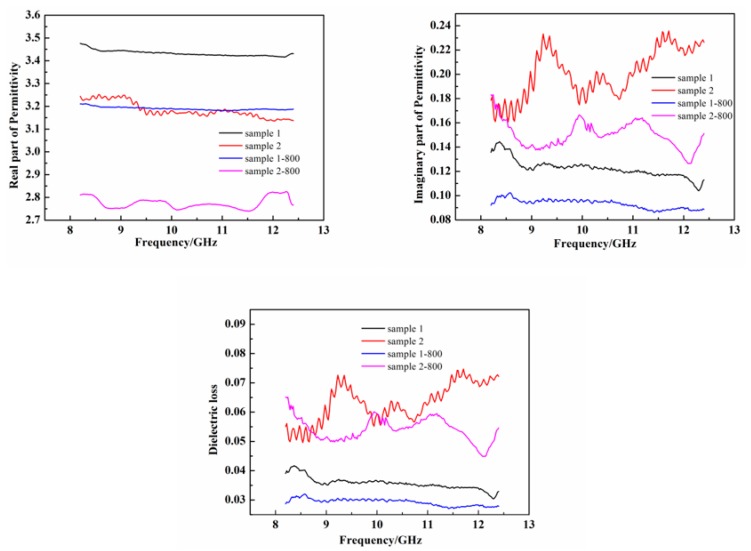
Dielectric properties of two fiber/resin composites in X-band, prior to and following annealing.

In addition to mechanical properties, the complex permittivity (ε = ε′ − jε″) is also an important performance parameter for wave-transparent applications. Figure 11 presents the real part (ε′) of permittivity, the imaginary part (ε″) of permittivity, and the dielectric loss (tan δ) of samples 1 and 2 as a function of frequency prior to and following annealing at 800 °C. The ε′, ε″ and tan δ at 10 GHz of sample 1 were 3.43, 0.12, and 3.6 × 10^−2^, respectively; for sample 2, the corresponding values were 3.18, 0.19, and 5.9 × 10^−2^, respectively. Following annealing at 800 °C, these results became 3.19, 0.03, and 9.6 × 10^−2^ for sample 1, respectively, whereas for sample 2, these results became 2.76, 0.16, and 5.6 × 10^−2^, respectively. The fiber/resin composites displayed a low increase of ε′ and ε″ compared to the epoxy resin (ε′_resin_ = 2.72, ε″_resin_ = 0.07), due to the conductive behaviors of the Si_3_N_4_ fibers. The permittivity of the fiber/resin composites, especially for Si_3_N_4_ fiber-H, presented an apparent frequency dependence, which was one of the features of a dielectric material [36].

It is known that the real part of permittivity is related to the polarization effects and that the imaginary part is related to the electrical conductivity [25,37]. In the Si_3_N_4_ fiber, the Si_3_N_4_ was a polar molecule, becoming the dipole under an applied electric field. Under that field, the dipolar polarized and the dipolar polarization was a relaxation, presenting long relaxation time and attenuating high amount of energy [24]. The Si_3_N_4_ fiber-L with a higher content of Si_3_N_4_ had a higher ε′, which implied that it possessed a stronger polarization ability.

For dielectric materials, the electrical conductivity (σ) is related to its dielectric property, which can be evaluated through the following equation [38]:

(8) $${\sigma \varepsilon }^{\prime \prime }\left(\omega \right)\;=\;{\omega \varepsilon }_{0}\varepsilon^{\prime \prime }(\omega ),$$

where σ is the electrical conductivity, ε_0_ is the free space permittivity (ε_0_ = 8.854 × 10^–12^ F/m), and ω is the angular frequency. The resistivity and conductivity values of both fibers, prior to and following annealing, are presented in Table 4. Compared to the Si_3_N_4_ fiber-L, the Si_3_N_4_ fiber-H had a higher conductivity, which might be attributed to the highly electrical conductive phase of SiN_x_O_y_ compared to the Si_3_N_4_. The relationships between the imaginary part and conductivity of two Si_3_N_4_ fibers corresponded reasonably with Equation (6). It is known that the dielectric constant and dielectric loss decrease as the porosity increases [39]. This was caused by the SiN_x_O_y_ decomposition following annealing at 800 °C. Consequently, both fibers annealed at 800 °C had lower dielectric constant and dielectric loss compared to the as-received fibers.

## 4. Conclusions

For the two types of Si_3_N_4_ fibers studied in this work, the microstructure, the mechanical properties and the dielectric properties were investigated prior to and following heat treatment at 800 °C under vacuum. The microstructural analysis demonstrated that both fibers consisted of amorphous Si_3_N_4_, as well as SiO_x_ and SiN_x_O_y_ phases. In addition, the Si_3_N_4_ fiber-H possessed a higher amount of SiN_x_O_y_ phase. Compared to the Si_3_N_4_ fiber-H, the Si_3_N_4_ fiber-L had a higher tensile strength and an improved thermal stability, as a result of its purer component. Following heat treatment, apparent defects and porous surface structures were noted in the Si_3_N_4_ fiber-H, leading to degradation due to the amorphous SiN_x_O_y_ phase decomposition. Moreover, the Si_3_N_4_ fiber-L had a higher permittivity and conductivity, resulting in its better polarization ability. Furthermore, the complex permittivity decrease of the annealed Si_3_N_4_ fiber/epoxy composites was also related to the SiN_x_O_y_ decomposition. The relatively higher strength, the better thermal stability and the same excellent dielectric properties indicated that the Si_3_N_4_ fiber-L possessed a high serving life at a serving temperature of at least 800 °C. This work could also contribute to the coordination of different service requirements and preparation processes and the selection of high-temperature wave-transparent materials for a potential application in ceramic matrix composites in harsh environments.

## Figures and Tables

**Table 1:** Fundamental characteristics of the silicon nitride (Si_3_N_4_) fibers.

Materials	Element/wt %				Diameter/μm
	Si	N	O	C	
Si_3_N_4_ fiber-L	59.79	36.0	3.56	0.65	12.2
Si_3_N_4_ fiber-H	52.39	33.14	13.56	0.91	13

**Table 2:** Elemental analysis of Si_3_N_4_ fibers following annealing.

Materials	Element/wt %			
	Si	N	O	C
Si_3_N_4_ fiber-L	66.97	27.23	5.12	0.68
Si_3_N_4_ fiber-H	64.26	25.68	9.24	0.82

**Table 3:** Comparisons of Weibull statistics and tensile strength of two Si_3_N_4_ fiber bundles annealed under vacuum.

Materials	Temperature/°C	Weibull Modulus	Scale Parameter/MPa	Tensile Strength/MPa
Si_3_N_4_ fiber-L	as-received	17.80	1137.9	1104.4
	800	13.31	1142.2	1098.7
Si_3_N_4_ fiber-H	as-received	13.92	866.8	835.0
	800	13.45	663.1	638.0

**Table 4:** Resistivity and conductivity of two fibers prior to and following annealing.

	As-Received		Following Annealing	
Materials	μ/Ώ m	σ/S m^−1^	μ/Ώ m	σ/S m^−1^
Si_3_N_4_ fiber-L	18.94	0.0528	24.29	0.0412
Si_3_N_4_ fiber-H	31.17	0.0321	33.39	0.0299
